# Do Parenting Styles Affect Children’s Oral Health in Saudi Arabia?

**DOI:** 10.7759/cureus.6002

**Published:** 2019-10-26

**Authors:** Maram A Alagla, Aljohara AL Hussyeen, Latifa Alhowaish

**Affiliations:** 1 Pediatric Dentistry, King Saud University, Riyadh, SAU

**Keywords:** parenting style, oral hygiene, dental caries, children, oral health

## Abstract

Objective

The purpose of the study is to correlate the parenting styles of parents with the oral health of their children, in Riyadh, Saudi Arabia.

Study design

Two hundred and eighty healthy preschool children, who have never been to the dentist, were recruited. Parenting style was determined by the Parenting Style and Dimensions Questionnaire (PSDQ). World Health Organization (WHO) criteria and simplified debris index (DI-S) were used for the diagnosis of dental caries and oral hygiene of the children respectively.

Results

Two parenting styles were identified among Saudi parents; authoritative (94%, n = 265) and permissive (6%, n = 17). The majority of children were brushing by themselves (n = 130, 46.1%) and once per day (n = 163, 57.8%). Significant correlations were detected between parenting style and children’s brushing times (P-value of 0.016) and the number of meals consumed by children (P-value of 0.031). The age of the child and oral hygiene score were significantly correlated to dental caries (P-value < 0.05).

Conclusion

Two parenting styles were identified among Saudi parents. Parenting style influenced the child’s oral health but not significantly. Early childhood caries and fair to poor oral hygiene were commonly detected among children. Clinically interesting correlations were identified regarding factors affecting the child’s oral health status.

## Introduction

Parentship is a special phase that individuals pass through and is full of positive and negative attitudes that parents can make in the process of raising their children. Family is the first encounter for the child to have a close relationship with, especially mothers [1]. Parenting style is an attitude that parents perform toward raising their children [2]. The growth of the child is directly influenced by the style of parenting and the surrounding home environment [3,4]. Parenting style as conceptualized by Baumrind (1971) provided a two-arm framework. These two arms or dimensions (responsiveness and demandingness) were a result of combining Baumrind’s parenting typology with previous definitions of parenting by Darling and Steinberg [4]. Parent responsiveness indicates the level a parent responds to his or her child’s needs and demands. It involves the warmth, supportiveness, self-assertion, and regulation and accepting the individuality of each child to become a healthy and mature adult [5]. Parent demandingness, on the other hand, is the efforts parents make to align the behavior of their children by guidance, supervision, and disciplinary acts to make the child’s behavior integrates with the family and society [4]. The balance between these two arms determines the style of parenting [3].

Parenting styles are known to be authoritative, authoritarian and permissive [4]. The authoritative parenting style is known for its high warmth and high demandingness qualities [5]. Authoritative parents do provide emotional support to their children and are responsive to their demands. They also set clear limits and provide an engaging communication [6]. The authoritarian parenting style (low warmth, high demandingness) is characterized by low sensitivity to the child’s opinion and autonomy and placing strict demands on the child without appreciating his/her ability to comprehend and mature [5]. This type of parenting does not involve warmth, understanding, and communication between parents and children. It also includes the use of directives and disciplinarian techniques. The authoritarian parent usually resolves the rules and the child is expected to follow them without resentment. The permissive parenting type (high warmth, low demandingness) exerts no control on the child’s behavior, demands no behavioral adjustment and limited child responsibilities and commands [3]. Permissive parents do not display any authority over their children and avoid the child’s confrontation.

As pediatric dentists, parenting style can manipulate the child’s oral health in many aspects that are of significance to the oral health of a child. These aspects include the family’s oral health practices and the dental caries level of the child. Studies have identified the existence of a relationship between the oral health of the child and the parenting style [7]. Dental caries is considered to be a multifactorial common and chronic oral disease [8]. It results from an interaction between four factors including carbohydrates, microorganisms, a susceptible tooth and time. Dental caries is highly prevalent among the children world-wide. It was reported by the WHO to be around 83.4% in lower-middle-income countries [9]. A recent prevalence indicated that 83% of Saudi children are affected by dental caries [10]. To investigate aspects that contribute to the endemic of dental caries among children, parenting style is one aspect. One study reported that authoritative parents had children with lower dental caries level compared to other parenting styles like permissive and authoritarian [7]. Similarly, parenting style was reported to affect the oral health of children. The oral health of mothers particularly influenced the children to acquire and practice the oral health practices whole their life [7,11]. Studies suggest that authoritarian and permissive parents are less likely to influence the importance of oral hygiene practices among their children [7,12].

Diet is another aspect of a child’s oral health. It was reported that authoritative parents were more responsible for the feeding options of their children and provided a healthier diet [13]. Collectively, these studies suggest that authoritative parents have better control over their children’s diet, oral hygiene practices and regular attendance for dental check-ups [7,12,14,15]. However, the evidence is still limited and further research is required especially in the region of Saudi Arabia. The purpose of this study was to investigate the correlation of parenting styles in Saudi Arabia with the child’s oral health at the child’s first dental visit.

## Materials and methods

Research ethical approval was obtained from the institutional review board (IRB) at the College of Medicine, King Saud University under the research number E-18-3045. The sample size was calculated using the formula: N = [(Zα + Zβ)/C]2 + 3, where C = 0.5 *In[(1+r)/(1-r)], assuming a correlation value of 0.20, with 90% power, and 0.05 level of significance, 259 study subjects are required, anticipating 20% incomplete questionnaire answers. The target sample size was 280 subjects [16]. Children were recruited in this study after meeting the following inclusion criteria: 1) Three- to six-year-old children attending the pediatric dentistry clinics of Dental University Hospital (DUH), King Saud University, Riyadh, 2) they have no medical condition and not diagnosed with any behavior or cognitive disease, 3) children must be visiting the dentist for the first time in their life for a check-up with no history of any previous dental emergency visit, and 4) living with both parents and willing to participate in the study. Children participated in this study were pre-screened on the computer system for eligibility, and parents were contacted by phone to assign an appointment. On the intervention day, the primary caregiver parent was asked to complete two questionnaires fully along signing a consent form; one questionnaire was regarding demographic data which was translated from English into Arabic and was validated and utilized in a previous study [17]. Minor modifications were added to the demographic questionnaire to suit the study objectives. The second questionnaire was the Parenting Style and Dimensions Questionnaire (PSDQ) [18]. After filling the questionnaires, parents and participants were guided to the clinic and were welcomed by the principal investigator and thanked to take part in the study. Questionnaires were confirmed and checked by the principal investigator for any missing information or misunderstood questions and efforts were made to explain unclear questions and to reach a suitable conclusion. Consent was checked before the child examination and the child was assented prior to his/her examination. To establish rapport with children, the examiner began with causal, warm-up questions, such as inquiries about the child’s favorite television cartoon. The dentist followed all the recommended personal hygiene instructions before examining the child after reviewing the medical and dental histories with parents [19]. The tell-show-do technique was followed to introduce the child to the examination kit [20]. Extra-oral and intra-oral examinations were carried out and teeth were counted for their completion. Prophylaxis was done using a slow speed handpiece set at the speed of 2500-3000 rpm, three small drops of prophylaxis paste were used and applied to all teeth via rubber cup [21]. A charting form was used to record the oral examination data. The d score of the caries index (dmfs) status was recorded by the dental assistant during the examination procedure. The oral hygiene index was recorded as recommended [22]. Acidulated phosphate fluoride (APF) 1.23% bubble-gum flavor was applied to upper and lower small-sized trays as recommended and post-operative instructions were given to the child and parent following the manufacturer’s instructions (Gelato Neutral PH gel, Keystone Industries, Gibbstown, NJ). The parenting style & dimension questionnaire (PDPQ), the short version, was utilized [18]. It contains 32 statements about different child-parent life interactions. The short yet reliable version of the PDPQ was used. The questionnaire assesses the parenting style based on Baumrind's parent’s typology: authoritative, authoritarian and permissive [3]. The parent was asked to rank the occurrence of each statement from (1 = never, 2 = once in a while, 3 = about half the time, 4 = very often, 5 = always). The rank from 1-5 is to display how often parents exhibited the behaviors mentioned in each question. The scoring key of the PDPQ was used to categorize parents into one of the three parenting styles. An overall mean score in each parenting style category was calculated and the highest score determined the parenting style of the parent. Permission was obtained from the author of PDPQ to translate the questionnaire into the Arabic language to be utilized in this study. The author provided the original version of the questionnaire and PDPQ was translated by a professional translator from the formal English version to the Arabic version through the back and forth translation [18]. The second questionnaire contained data about the child and the family inquiries about medical and dental health information, oral hygiene and dietary information, household income and parent’s educational level. The dental anxiety for parents was asked to parents along with the demographic questionnaire. This question is a single item question concerning the parents. The dental anxiety question (DAQ) has been used to indicate dental anxiety in several previous researches and displayed good validity [23,24].

Statistical analysis

The data collected from parents were matched between the parent/caregiver and the child information and clinical scores. Data were coded, entered and analyzed using Statistical Package for Social Science (SPSS) version 22 (IBM Corp., Armonk, NY) statistical software. Descriptive statistics (frequencies, percentages, mean and standard deviation) were used to describe the categorical and continuous variables. Shapiro-Wilk test was used to check for variable normality (P-value ≥ 0.05 indicates a normally distributed continuous variable). A chi-square test was used to analyze the relationship between parenting style and different child and parent factors. Simple linear regression analysis was used to identify factors affecting the child’s dental caries, while simple logistic regression analysis was used to assess factors affecting the child’s oral hygiene. Any variable with a P-value of < 0.25 was entered into multiple regression analyses to identify factors that best predicted the dependent variables in the study population, using beta values as a measure of the relative impact of each predictor on the outcome variable. A P-value < 0.05 was considered statistically significant [25]. The PDPQ questionnaire’s internal consistency (reliability) was assessed using Cronbach’s alpha coefficient (α) for each dimension and for the total questionnaire. Cronbach’s alpha coefficient values ≥ 0.70 were considered satisfactory.

## Results

General characteristics of the study participants and their parents

**Figure 1:**
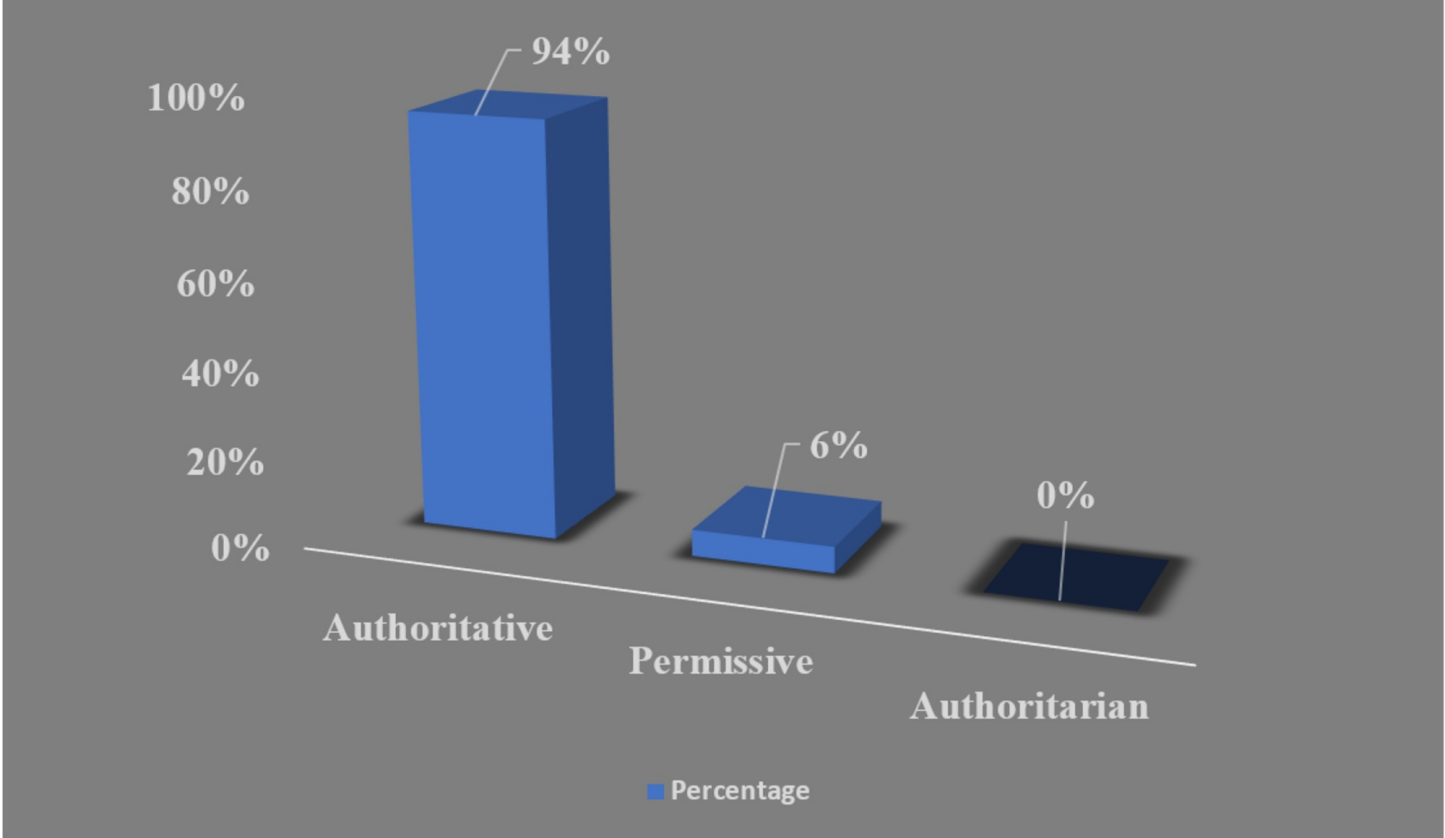
Classification of recruited parents based on their parenting style, n = 282

**Figure 2:**
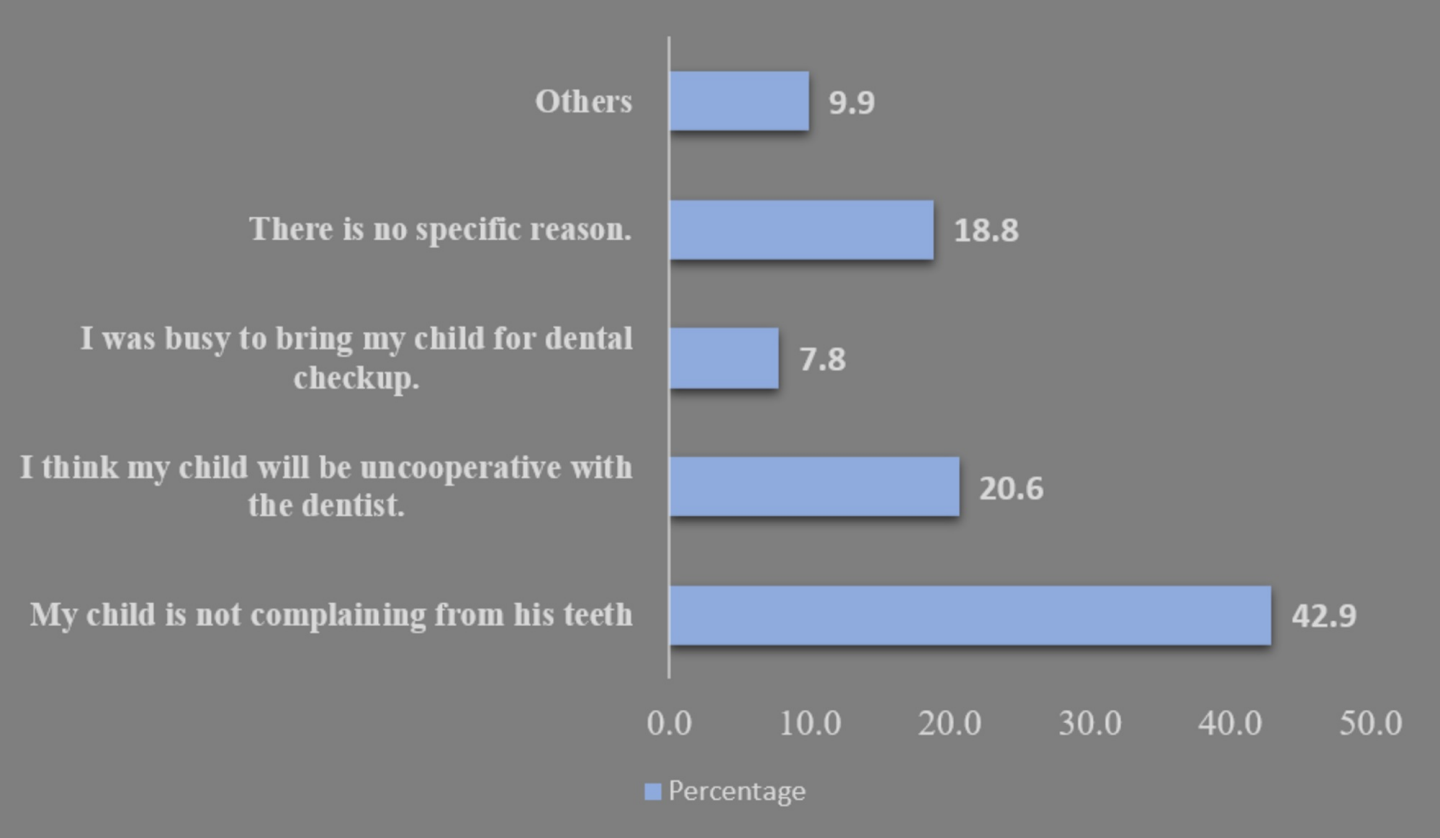
Reasons why children never visited a dentist, (n = 282)

Most of the parents were reported to adopt an authoritative parenting style (n = 265, 94%), while the permissive parenting style accounted for 17 parents (6.0%) (Figure 1). The socioeconomic level of the participating families was mainly a middle-middle-income class (n = 139, 39.4%). The majority of parents had a diploma or university degree (n = 161, 57.1%) for fathers and (n = 174, 61.7%) for mothers. Children were accompanied by their mothers predominantly who were responsible to fill the questionnaire (n = 211, 74.8%) (Table 1). Primarily 242 of children (85.8%) were weaned from bottle-feeding and the weaning age was mostly at the age of two years by 44% (n = 124) (Table 2). Children in the sample never had a previous dental check-up and most of the parents explained that the reason is that their children had no teeth complaint (n = 121, 42.9%) (Figure 2) (Table 1).

**Table 1:** General characteristics of the parents of the participants (n = 282) **Gender of parents who filled the questionnaire, *SAR (Saudi Riyal) = 0.266 USD

Variable		n (%)
Family income	Less than 3,800 SAR*	19 (6.7)
	From 3,900 to 7,700 SAR	88 (31.2)
	More than 7,700 to 22,900 SAR	139 (39.4)
	More than 22,900 to 38,200 SAR	33 (11.7)
	More than 38,200 SAR	3 (1.1)
Gender of parents**	Female	211 (74.8)
	Male	71 (25.2)
Father education	High school degree or less	84 (29.8)
	Diploma or University degree	161 (57.1)
	Postgraduate degree	37 (13.1)
Mother education	High school degree or less	77 (27.3)
	Diploma or University degree	174 (61.7)
	Postgraduate degree	31 (11.0)
Parent’s dental anxiety	Yes	123 (43.6)
	No	159 (56.4)

**Table 2:** General characteristics of the study participants (n = 282)

Variable		Mean ± SD	n (%)
Gender of child	Female		152 (53.6)
	Male		130 (46.1)
Age of child	Years	4.1 ± 1.0	
Does your child still on bottle-feeding?	Yes		40 (14.2)
	No		242 (85.8)
When the child was weaned?	Less than two years of age		56 (19.9)
	At two years of age		124 (44.0)
	More than two years of age		102 (36.2)
Does your child go to the nursery or preschool?	Yes		130 (46.1)
	No		152 (53.9)
Does the child visit a doctor for any medical condition?	Yes		0 (0.0)
	No		100 (100)
Does the child take any chronic medication?	Yes		0 (0.0)
	No		100 (100)

Dental care and dietary behavior adopted by the study participants

Regarding the oral hygiene of the children, the majority of the samples were brushing by themselves (n = 130, 46.1%). More than half of the children in the present study were brushing once per day (n = 163, 57.8%). More than three-quarters of the children (77.3%) consumed three meals per day. The consumption of sweetened beverages and sweets was high among the study participants with a mean consumption of more than six times per week (Table 3).

**Table 3:** Dental care and eating behavior adopted by study participants (n = 282)

Question		Mean ± SD	n (%)
Who cleans the child's teeth?	Child himself		130 (46.1)
	Mother		102 (36.2)
	Not brushing his teeth		50 (17.7)
How many times the child’s teeth are brushed daily?	Never		50 (17.7)
	One		163 (57.8)
	Twice		62 (22.0)
	Three times or more		7 (2.5)
How many main meals does the child eat daily?	1 – 2 meals	2.8 ± 0.6	64 (22.7)
	3 – 4 meals		218 (77.3)
How many times does the child drink sodas or sweetened juices per week?	0 – 2 times	6.4 ± 5.7	97 (34.4)
	Three or more times		185 (65.6)
How many times does the child eat sweets?	0 – 2 times	8.0 ± 5.9	52 (18.4)
	Three or more times		230 (81.6)

Child clinical examination characteristics

**Figure 3:**
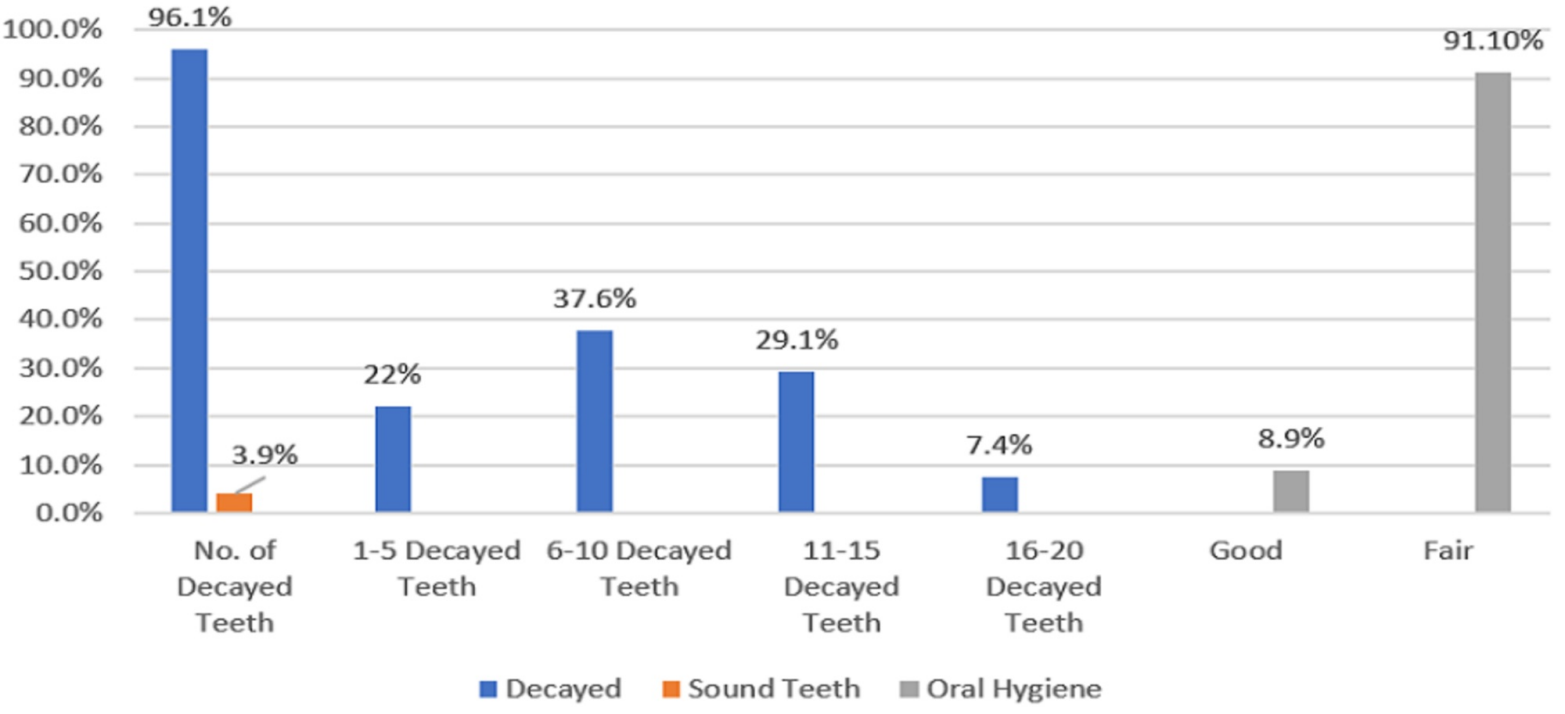
Clinical examination characteristics of the children, n = 282

The mean number of decayed teeth was about 8.8, SD ± 4.6. Most of the children (96.1%) presented with dental caries (n = 271). The degree of dental caries among the sample was mostly 6 to 10-decayed teeth (37.6%, n = 106). Regarding oral hygiene, 91.1% (n = 275) presented a fair/poor oral hygiene (Figure 3).

Parenting style and children’s oral health status and practices

The oral hygiene of the child was not significantly correlated to the parenting style (P-value 0.379). Furthermore, some children of authoritative parents (n = 25, 9.4%) had good oral hygiene compared to none of the children of permissive parents. The correlation between parenting style and dental caries experience among children was not significant. However, 4.2% of authoritative parents had children with caries-free teeth compared to none of the permissive parents (Table 4).

**Table 4:** Parenting style, dental caries and oral hygiene scores among children, n = 282 P-value < 0.05, using Chi-square test and Fisher’s exact test.

Variable		Parenting Style		P- Value
		Permissive N (%)	Authoritative N (%)	
Decayed teeth	No caries	0 (0)	11 (4.2)	1.00
	Caries	17 (100)	254 (95.8)	
	Zero decayed teeth	0 (0)	11 (4.2)	
	1 – 5 decayed teeth	1 (5.9)	61 (23)	
	6 – 10 decayed teeth	7 (41.2)	99 (37.4)	
	11 – 15 decayed teeth	7 (41.2)	75 (28.3)	
	16 – 20 decayed teeth	2 (11.8)	19 (7.2)	
Oral hygiene	Good	0 (0)	25 (9.4)	0.376
	Fair	17 (100)	240 (90.6)	

Brushing times were significantly related to parenting style with a significance P-value of 0.016, authoritative parents reported that their children were brushing more than twice per day (Table 5). The number of meals consumed by children was significantly related to parenting style (P-value of 0.031), with higher meal consumption among children of authoritative parents. The child’s sweet intake and sweet beverages consumption were not correlated to parenting style, nevertheless, more children of permissive parents consumed sweets and sweet beverages more than three times per week compared to authoritative parents (Table 5).

**Table 5:** Parenting style, oral hygiene and dietary practices *P-value < 0.05, using Chi-square test

Variable		Parenting Style		P- Value
		Permissive N (%)	Authoritative N (%)	
Brushing times	0 – 1	17 (100)	196 (74)	0.016*
	2 and more	0 (0)	69 (26)	
Brushing assistance	Child	10 (66.7)	120 (55.3)	0.434
	Mother	5 (33.3)	97 (44.7)	
Number of meals	1 – 2	8 (47.1)	56 (21.1)	0.031*
	3 – 4	9 (52.9)	209 (78.9)	
Sweet intake	0 – 2 per week	1 (5.9)	51 (19.2)	0.328
	3 or more per week	16 (94.1)	214 (80.8)	
Sweet beverage	0 – 2 per week	3 (17.6)	94 (35.5)	0.188
	3 or more per week	14 (82.4)	171 (64.5)	

Factors affecting the child dental caries level

To study the factors affecting the child’s dental caries level, the univariate linear regression analysis was used referring to a P-value of <0.25 as a significant value (Table 6). The factors, which influenced the dental caries experience of children as seen in Table 6, are the following: the age of the child, the socioeconomic factors including total family income and parent’s education, and the oral hygiene level of the child; moreover, the weaning from bottle-feeding, the parent’s dental anxiety and the parenting style. The significantly influencing factors mentioned above were further analyzed by the multiple linear regression model referring to a P-value of < 0.05 as a significance level (Table 7). The age significantly affected the dental caries experience of the child with a P-value of 0.028, the older the child the more dental caries was observed. The parental dental anxiety was significantly reported to affect the child’s dental caries level (P-value of 0.031), the more anxious the parent was the less the dental caries experience of the child. Finally, the oral hygiene level was significantly correlated to the dental caries status of the child, the less the oral hygiene was the more the dental caries was detected by a P-value of 0.003 level of significance.

**Table 6:** Univariate linear regression analysis for factors affecting children’s dental caries, (n = 282) #SAR (Saudi Riyal) = 0.266 USD *P-value < 0.25 **B estimate = Beta coefficient ***Beta = Standardized regression coefficient ****R2 = Coefficient of determination

Variable		Dependent variable: Number of decayed teeth				
		B Estimate**	SE	Beta***	R2 %****	P-value
Gender of child	Female	Reference -0.807	0.335	-0.078	0.6	0.145*
	Male					
Age of child	3-4 years	Reference 1.522	0.570	0.158	2.5	0.008*
	5-6 years					
Family income	7,700 SAR# or less	Reference -1.401	0.564	-0.147	2.2	0.014*
	More than 7,700 SAR					
Father education	High school degree or less	Reference -1.503	0.598	-0.149	2.2	0.013*
	Diploma or university degree or higher					
Mother education	High school degree or less	Reference -1.573	0.614	-0.151	2.3	0.011*
	Diploma or university degree or higher					
Does your child still on bottle-feeding?	No	Reference -1.242	0.789	-0.094	0.9	0.117*
	Yes					
Parents anxiety	No	Reference -1.214	0.553	-0.130	1.7	0.029*
	Yes					
Parents parenting style	Permissive	Reference -2.127	1.155	-0.109	1.9	0.067*
	Authoritative					
Oral hygiene	Good	Reference 2.772	0.959	0.170	2.94	0.004*
	Fair					

**Table 7:** Multiple linear regression analysis for factors affecting children’s dental caries, (n = 282) #SAR (Saudi Riyal) = 0.266 USD *P-value < 0.05 **B estimate = Beta coefficient ***Beta = Standardized regression coefficient ****R2 = Coefficient of determination

		Dependent variable: Number of decayed teeth				
Variable		B Estimate**	SE	Beta***	R2 %****	P-value
Age of child	3-4 years	Reference 1.248	0.566	0.129	1.7	0.028*
	5-6 years					
Parents anxiety	No	Reference -1.175	0.543	-0.126	1.6	0.031*
	Yes					
Oral hygiene	Good	Reference 2.819	0.931	0.173	3.0	0.003*
	Fair					

Factors affecting child oral hygiene

The factors affecting the oral hygiene of the child in this study are presented using univariate logistic regression and considering a P-value of <0.25 as a significant value as seen in Table 8. These factors are: teeth brushing, assistance during teeth brushing and brushing times per day. Since multicollinearity was detected between who cleans the child teeth variable and number of times the child brushes his/her teeth variable (correlation coefficient >0.9), one of the variables was removed before conducting multiple logistic regression analysis. Therefore, only two variables were analyzed by the multiple logistic regression analysis (did the child brush his/her teeth and the number of times the child brush his/her teeth) and no significant relationship was observed between the above-mentioned factors and the child’s oral hygiene (Table 9).

**Table 8:** Univariate logistic regression analysis for factors affecting children’s oral hygiene, (n = 282) *P-value < 0.25

		Dependent variable: Child oral hygiene (good oral hygiene coded 0, fair oral hygiene coded 1)				
Variable		B estimate	SE	P-value	Odds ratio	95% CI for OR
Brushing teeth	No	Reference -1.732	1.033	0.094*	0.2	0.0-1.3
	Yes					
Who clean child's teeth	Himself	Reference -0.592	0.456	0.195*	0.6	0.2-1.4
	The mother					
Number of times the child brushes his/her teeth	0-1 per day	Reference -0.805	0.434	0.064*	0.5	0.2-1.0
	2 or more per day					

**Table 9:** Multiple logistic regression analysis for factors affecting children’s oral hygiene, (n = 282) *P-value < 0.05

		Dependent variable: Child oral hygiene (good oral hygiene coded 0, fair oral hygiene coded 1)				
Variable		B estimate	SE	P value	Odds ratio	95% CI for OR
Brushing teeth	No	Reference -1.572	1.048	0.145	0.2	0.0-1.7
	Yes					
Number of times the child brushes his/her teeth	0-1 per day	Reference -0.590	0.442	0.182	0.6	0.2-1.3
	2 or more per day					

## Discussion

Parenting style and oral hygiene

Children’s oral hygiene was found to be not significantly associated with the type of parenting. This finding could be related to the fact that children come to the dental screening visit after school where they usually consume some snacks and exhibit dental plaque during a dental examination. The finding is parallel to a previous study that described a similar observation [12]. Oral hygiene is a multifactorial result and parenting style is one factor. Other demographic factors have an influence on the oral hygiene of the study participants. In this study an interesting finding was observed: children of authoritative households had good oral hygiene (9.4%) compared to none of the children of permissive parents. Moreover, children’s brushing times were significantly related to parenting style as 26% of authoritative parents reported that their children were brushing more than three times per day compared to none of the permissive parents. This finding supports the literature that authoritative parents have better control over their children’s oral hygiene practices including tooth brushing [7, 12]. The current study did not identify any significant relationship between parenting style and child’s brushing assistance and this could be attributed to the fact that most of the study participants were brushing by themselves so the difference was not significant. Our findings concur with the literature that authoritative parents have better control over their children’s diet and oral hygiene practices. Hence, children of authoritative households manifest a better oral hygiene status compared to other parenting styles [7, 12].

Parenting style and child’s dental caries

Correlation between dental caries and parenting style was not significant, it could be related to the fact that dental caries as oral hygiene is a multifactorial process so parenting is one factor among other distinct factors. The other explanation could be that Saudi children had increased dental caries and the current study agrees with previous studies [10]. The increased dental caries experience among Saudi children could be due to the high consumption of cariogenic diet, the lifestyle of the Saudi population and the lack of effective tooth brushing. However, an interesting finding was that children who had all sound teeth came from authoritative homes which correspond to other studies reporting that children of authoritative parents had less dental caries experience compared to children of other parenting styles [7]. This could be attributed to the fact that authoritative parents have better control over their children’s diet and they guard the access of children to the cariogenic diet. Authoritative parents are more likely to keep track of their children’s dental checkup and apply better oral hygiene practices [15]. On the contrary, permissive parents are more forgiving about their children’s behavior including sugar consumption. They are less likely to control the access of their children to the cariogenic diet if they are not the one providing it to bribe the child to control his/her misbehavior [26].

When factors affecting dental caries were studied further, our study reported that age, parental dental anxiety, and oral hygiene affected the child’s dental caries experience. The present study indicates that the older the child is the worse the dental caries experience. It is attributed to the fact that older children had their teeth erupted longer than younger children thus are more exposed to caries due to time factors besides more exposure to cariogenic food. A compelling finding is that parental dental anxiety was inversely correlated to dental caries experience of the child. Anxious parents had children with fewer dental caries which could be interpreted by the fact that anxious parents seek dental care in the early stages of the caries process to prevent their child to be exposed to painful experience. Anxious parents may demand frequent oral hygiene practices from their children to avoid dental treatment as possible. Our study reported that children with improved oral hygiene developed fewer dental caries than children with worse oral hygiene. It is a causality result, children who brush more have better oral hygiene and fewer dental caries and our study supports the literature [27]. The observed data presented in the current study on the prevalence of dental caries among children of Saudi Arabia is substantially below the targeted global goal of WHO to decrease the burden of dental caries by 50% in the year 2000. Additionally, the burden does not meet the WHO (World Health Organization) and FDI (Fédération Dentaire Internationale) 2020 oral health global goal and is not nearly met [28]. The purpose of these goals was to guide health care policymakers to improve the oral public health of the nation. However, the absence of baseline data in the developing world challenges the attainment of WHO goals. This study collectively with previous studies confirms the endemic nature of oral disease among children of Saudi Arabia [10, 29].

Parenting style and child’s dietary habits

Authoritative parents reported that their children consumed more main meals than permissive parents did and this finding confirms the fact that parenting style affects the diet of children. Authoritative parents as explained previously have better control over their children’s diet, meal consumption and meal times. Therefore, the literature suggests that authoritative homes have certain rules regarding the regularity and the quality of the child’s diet and the current study affirms with this fact [13]. However, children's intake of sweet food and beverages was high among all the study participants but permissive parents reported that their children consumed sweets more than three times per week more than authoritative parents did. The high sweet intake is expected due to the type of lifestyle among the Saudi population. Saudi children have higher access to sweets since it is sometimes provided in schools and daycares. Our study coincides with a previous study on Saudi school children who were found to have a higher tendency towards consuming sugar-rich food and the sweet consumption increased with age [30].

Multiple findings indicated a lack of dental knowledge and dental knowledge application among parents. Fifty children in our study reported not brushing their teeth, most of the study participants were brushing by themselves without assistance and most of the children were brushing once per day. Oral health habits practiced by the study participants were less than the recommendation of the WHO in 2013. Dental health education should be encouraged more through media, schools, hospitals and dental public health efforts. When parents in our study were asked about the reason for not taking the child for a dental check-up previously they mostly answered that their child was not complaining from his/her teeth. This indicates multiple facts: lacking in the dental knowledge among Saudi parents who link visiting the dentist to only cases of emergency. Additionally, some parents identified the importance of primary teeth but its importance is incomparable to the importance of permanent teeth, which requires them to take their children to emergency visits only. Some parents identified verbally that access to dental treatment was not easy and providing dental treatment to their children in private practice was financially beyond their capability unless it is urgent. Therefore, attention from the ministry of health toward focused and frequent implementation of dental education programs to expecting mothers, primary health care centers, preschools and schools is needed. Moreover, it is needed to provide annual or semi-annual visits to preschools and primary health care centers to provide dental prevention programs including, fluoride application, oral hygiene instructions, and prophylaxis.

## Conclusions

Two parenting styles of three main types were identified among Saudi parents including authoritative and permissive parenting styles. Parenting style did not affect the oral hygiene and the dental caries experience of children directly. However, a slight correlation is detected and addressed. Parenting style influenced the dietary behaviors of children yet the consumption of sugar was high among all the study samples. Early childhood dental caries is confirmed to still be highly detected among Saudi children. Fair and poor oral hygiene scores were common among preschool children.

## Figures and Tables

**Figure 4:**
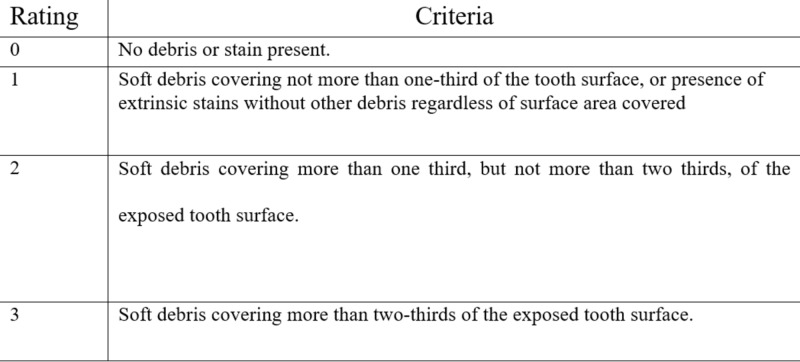
Simplified oral hygiene index-simplified debris index

## Appendices

Parenting Styles and Dimensions Questionnaire (English-short-version)

Please make a rating for each item: How often you exhibit this behavior as parents, or as a single parent

“I” or “We” Exhibit This Behavior

1=Never 2=Once in a While 3=About Half the Time 4=Very Often 5=Always

1. I am responsive to our child’s feelings and needs.___________

2. I use physical punishment as a way of disciplining our child______

3. I take our child’s desire into account before asking the child to do

something__________

4. When our child asks why they must conform, I state: “because I said so,”

Or “I am your parent and I want you to________

5. I explain to our child how we feel about the child’s good and bad behavior_________

6. I spank when our child disobeys__________

7. I encourage our child to talk about his/her troubles __________

8. I find it difficult to discipline our child___________

9. I encourage our child to freely express himself/herself even when disagreeing with parents________

10. I punish by taking privileges away from our child with little to no explanation______

11. I emphasize the reasons for rules __________

12. I give comfort and understanding when our child is upset___________

13. I yell or shout when our child misbehaves__________

14. I give praise when our child is good________

15. I give into our child when the child causes a commotion about something _________

16. I explode in anger towards our child ___________

17. I threaten our child with punishment more often than actually giving it ___________

18. I take into account our child’s preferences in making plans for the family _________

19. I grab our child when being disobedient _________

20. I state punishments to our child and do not actually do them_______

21. I show respect for our child’s opinions by encouraging our child to express them

22. I allow our child to give input into family rules __________

23. I scold and criticize to make our child improve __________

24. I spoil our child ___________

25. I give our child reasons why rules should be obeyed

26. I use threats as punishment with little or no justifications__________

27. I have warm and intimate times together with our child_________

28. I punish by putting our child off somewhere alone with little if any explanations ____

29. I help our child to understand the impact of behavior by encouraging our child to talk about the consequences of his/her own action______

30. I scold or criticize when our child’s behavior does not meet our expectations_______

31. I explain the consequences of the child’s behavior ___________

32. I slap our child when the child misbehaves _________

Simplified oral hygiene index-Simplified debris index

                  Demographic Questionnaire- English

 Child Serial number

Child Sex:           1. Male           2. Female

Filled by:              1- Mother      2- Father

1.     Does the child currently visit a doctor for reasons other than teeth?

A. Yes      B. No                  If yes, kindly mention the reason:…………….

2. Does the child currently take any medicines?

A. Yes      B. No         If yes, Kindly mention the name of the medicine:………..

3. The reason for not visiting the dentist until now:

A. He/she never complained    B. I don’t think he/she will cooperate

C. I am very busy to take him to the dentist     D. No reason

E. Other reasons:……………..

4. Does your child go to the Nursery or preschool?       A. Yes      B. No

5. Are the child's teeth brushed at home?         A-Yes    B-No 

  If yes, C.  Who cleans the child's teeth: .......................          

6. How many times the child's teeth are brushed daily? 

1-once               2-Twice                3-Three times or more

7. How many main meals does child eat daily?   (            )

8. How many times does the child drink sodas? (        /Day/Week/Month)

9. How many times does the child eat sweets? (         /Day/Week/Month)

10. Do your child still on bottle-feeding?     1.Yes    2.No

11. When the child was weaned? 

 1. Less than 2 years of age   2. 2 years          3. More than 2 years of age

12. What is the educational level of the child's father?

1. Secondary School or below

2. Two years diploma

3. University Degree

4. Higher than a University Degree

13. What is the educational level of the child's mother?

1. Secondary School or below

2. Two years diploma

3. University Degree

4. Higher than a University Degree

14. Family's total income?

A. Less than 3,800 Riyals

B. From 3,900 to 7,700 SAR

C. More than 7,700 to 22,900 SAR

D. More than 22,900 to 38,200 SAR

E. More than 38,200 SAR

15. Do you have dental anxiety?  

A. No. 

B. A little.  

C. Yes, quite.

D. Yes, I get very anxious when I visit the dentist.

English Consent

Introduction:

Your dear child is invited to participate in the current study, which aims to correlate the relationship between parenting style with the child oral health.

You can take whatever time you need to decide if your child is going to join the study, you can also discuss the study with your family, friends, doctors or anyone else that you trust, it is very important to be fully aware of your decision and it is our pleasure to help you as much as we can to make your right decision.

What does the study involve?

If you agree to let your child join this study, your child will be called for an appointment at the University Dental Hospital (UDH) at King Saud University; your child will have access to the hospital and a file number and record.

The child will be examined and prophylaxis will be done for him/her and you will be asked to kindly fill a questionnaire related to your child and another questionnaire that is related to you.

The clinician and the investigator may decide to stop the study or your child's participation at any time they judge it is for your child’s benefit. You can also stop your participation at any time and you will not lose any benefits.

Risks:

There are no known risks expected from taking part in this study.

Benefits:

The following benefits are expected if your child took part in this study:

- The information taken from your child will add to the knowledge of the current knowledge of the parenting styles practiced in Saudi Arabia and how it affects the children’s oral health. The community will benefit from such knowledge by finding proper approaches to improve child oral health.

- Your child will also have their file, treatment, and follow-up under the care of UDH.

Confidentiality:

Your child's information will be protected from unauthorized disclosure; your child will be given a specific research number. The data will be saved on the computer of the investigator and she is the only one who will be exposed to the child or family information.

I have read the above-mentioned information or it has been read to be. I had a chance to ask any questions and my questions were answered to my satisfaction. I consent voluntarily for my child to be enrolled in this study.

*If the parent or guardian is illiterate:

The consent was read to the parents or guardian and questions were answered to their satisfaction, they agreed to sign the consent voluntarily.

Name of the participating child: _______________________

Name of the parent/ guardian: _______________________

Signature of the parent/ guardian: ____________________

Date: _______________

Investigator statement:

I consent that I have read and explained the study consent form for the child’s parent or guardian up to their full comprehension and that their inquiries were fully addressed and answered. I consent that the guardian was given time to ask, decide and sign. I consent that they signed voluntarily without any forces.

Name of the investigator: _____________________

Signature of the investigator: ____________________ Date: ______
